# Age matters: health, older people and gerohygiene in the late Soviet Union

**DOI:** 10.1017/mdh.2022.17

**Published:** 2022-07

**Authors:** Susan Grant

**Affiliations:** Liverpool John Moores University, John Foster Building, 89–98 Mount Pleasant, Liverpool L3 5UZ, UK

**Keywords:** Older people, Health, Soviet, Physical culture, Labour, Gerontology

## Abstract

In the late Soviet period, a great deal of research was conducted on older people’s health, with the Institute of Gerontology Academy of Medical Sciences (AMN) USSR in Kyiv spearheading a great deal of this. Of particular interest was older people’s ability to work beyond retirement age, the issue of premature ageing, as well as physical activity, diet and living conditions. Many of these interests came under the concept of ‘gerohygiene’, which also reflected the Soviet Union’s prophylactic approach to eldercare (and healthcare more generally). Discussions about older people and Soviet research on gerohygiene are important for furthering our understanding of ideas around healthy ageing and the Soviet project more generally. The Soviet, and indeed socialist, research on gerohygiene sheds light on ideas around active ageing, premature ageing and work practices for older people. It also shows that the role of old people belonged to the wider Soviet effort of contributing to the communist project and shaping society. In this article, I define and examine the broad concept of ‘gerohygiene’ and then assess how gerohygiene applied to older people’s health in relation to both physical activity and labour.

In 1966, the head of the Kyiv Institute of Gerontology, D. F. Chebotarev, wrote:

‘The last ten years has seen the successful development of a new branch of gerontology – gerohygiene, a task that touches on questions of occupational hygiene, diet, motor activity (*dvigatel’naia rezhima*), and other problems of hygiene for older and elderly people’.^1^

In this article, I focus on two aspects of Soviet gerohygiene in the late socialist period: physical activity and labour. I show how these were interconnected in their relationship to older people’s health and that the Soviet state came to see gerohygiene as a way of forging healthy older citizens who would continue to participate in the construction of communism after they had officially retired from work. Although only a fraction of a vast research programme, Soviet work on gerohygiene was an important part of the Soviet gerontological agenda. Gerohygiene research highlights the Soviet prophylactic approach to healthcare and an interest in finding ways to mobilise people to take charge of their own health.

In a world with an ageing population, we would be remiss not to examine and learn from Soviet work on gerohygiene (or, indeed, broader socialist work on gerohygiene). These are aspects of ageing that can be read as modes of preservation and transformation that are just as relevant today. Soviet ideas around gerohygiene aimed to promote health, longevity and labour ability but some of the activities and outcomes associated with it also provided older people with agency, autonomy and empowerment, even though these were not the goals of the Soviet state. The transformative and prophylactic dimensions of gerohygiene chimed with the Soviet Union’s longstanding interest in shaping the individual and making people into ideal socialist citizens. Recent work on Soviet subjectivities, especially in the 1920s and 1930s, shows how some Soviet people attempted to mould themselves into the type of citizen that the Soviet state promoted through education, labour, and political literacy.^2^ Although the youth of the 1920s were becoming old women and men in the late socialist years, they were still part of a project of both preservation and transformation. As the population aged and the numbers of those suffering from cardiovascular, neurological, oncological and other age-related diseases increased, researchers investigated ways of preserving middle-aged and older people’s health and, *ipso facto*, attempted to reinforce the legitimacy of the socialist project where life was to have become better and happier.^3^

Interest in older people came at a time when the Soviet Union was undergoing a process of destalinisation, and when Nikita Khrushchev (1956–64) and later Leonid Brezhnev (1964–82) pursued policies that attempted to raise living standards through a range of welfare reforms.^4^ Older people – primarily pensioners aged over 45 years (the retirement age was 55 for women and 60 for men) – have not featured much in research on the late Soviet period, and yet older people and their social, medical and cultural needs informed policy and planning in the late Soviet period. If the younger Soviet generation flocked to universities to study science, answered Khrushchev’s call to conquer the Virgin Lands or became drawn to counterculture, members of the older generation were grappling with their deteriorating health while still tethered to the wider socialist goal of participating in society.^5^ Older people might not have made headlines or received much historical attention to date, but the late Soviet period was defined as much by its ‘grey’ generation as it was by its youth.

The Soviet Union was not alone in having an ageing population or an interest in gerontology. Ageing was and is a global issue. The Soviet experience should be read as part of a broader international gerontological story, and I draw on comparisons where relevant. Although the USA and the United Kingdom also had their research centres and institutes for the study of gerontology, the integrated and in-depth nature of the Kyiv-based Institute of Gerontology’s research on the role of exercise, health and ageing arguably set the Soviet Union apart.^6^ Scholars of gerontology today have, however, often overlooked the Soviet and socialist contribution to the development of international gerontology due to their ideological and political marginalisation during the Cold War.^7^ But the Soviet experience needs to be included in broader histories of gerontology and ageing. In its examination of gerohygiene, this article shows that gerontological research in the USSR has valuable lessons about older people’s contribution to labour and society, and about physical activity as a form of health preservation as people grow older.

## Methodology and literature

A rich collection of reports, correspondence and plans from the Institute of Gerontology are archived in the ‘Central State Archives of the Supreme Organs and Administrations in Ukraine’ (TsDAVO) while the Institute of Gerontology’s library in Kyiv houses a vast collection of books and articles on gerontology and geriatrics. By dint of the Institute’s location in Kyiv, much of the research is centred on Ukraine, but that did not mean that wider Soviet interests were not represented. As part of the Academy of Medical Sciences, the Institute had responsibilities that extended to the Soviet Union as a whole and so it worked with both central and local actors. Indeed, correspondence shows that researchers there regularly engaged with colleagues across the Soviet Union and internationally. That said, the Ukrainian republic was an important centre of medical and scientific research. Moscow often looked to Kyiv for examples of medical leadership and innovation.

The recent work of philosopher Martha Nussbaum and surgeon and writer Atul Gawande has helped to raise the issue of ageing and mortality in the public consciousness, but there is much that we can learn from history.^8^ Similarly, in his recent article on ageing in East and West Germany in the 1950s, historian James Chappel has echoed Nussbaum’s concern that the study of old age, though ‘in vogue in the 1980s and early 1990s’, has since languished at the margins of historical attention.^9^ Rather than rewriting history anew, scientists, medical workers and the general public can learn important lessons from the past.

The first major work to address the history of ageing was Peter Stearns’ 1976 book, *Old Age in European Society.* ^10^ Stearns’ work was followed by that of scholars such as Carole Haber, Thomas R. Cole, Brian Gratton, Chris Phillipson, Haim Hazan, Howard P. Chudacoff and Andrew Achenbaum – scholars who have produced excellent work on the history of ageing.^11^ Still, the emphasis remains on the USA and Western societies, with scant attention paid to Soviet approaches to ageing.

A handful of historians have published on Soviet ageing and gerontology: in his work on early Soviet Russia, Stephen Lovell focuses on pensions over the entire Soviet period and highlights intergenerational connections, particularly in urban centres as well as the precarious nature of entitlement to a pension.^12^ In ‘Soviet Socialism and the Construction of Old Age’, Lovell examines medicalisation and welfare as two key issues that affected the lives of older people and their ability to access pensions.

Lukas Mücke’s monograph, published in 2013, is a study of pension reforms between 1956 and 1972 (the first reform was in 1956 and the second in 1964).^13^ Mücke’s work provides a long overdue analysis of old age issues in the Soviet Union, with a focus on economic and social history. Both Mücke and Lovell draw on Mark Edele’s concept of ‘entitlement community’, finding similarities between Second World War veterans and older people in their quest to gain access to social welfare and state recognition as a community.^14^

This is also the case in Steven Harris’s article about older people’s experience of getting access to housing under Khrushchev and Brezhnev. Harris shows that older people in the 1950s and 1960s – the generation that came of age under socialism – believed they were entitled to better living conditions. They helped construct communism, survived the Terror (1937–38) and defeated the Nazis, and consequently, Harris argues, wanted decent housing in return.^15^ In their work on Soviet pensioners and socialist activism in the Khrushchev era, Alissa Klots and Maria Romashova argue that older people and especially female pensioners helped shape Soviet society through their volunteer work in public organisations. Their reading of older people’s public activism in the Khrushchev period (1956–64) shows how older people’s understanding of ‘communism was constantly evolving’.^16^ An analysis of gerohygiene and elder health furthers our understanding of older people in the late socialist period and the larger Soviet project to build communism.

## Defining Soviet gerohygiene

Russian and Ukrainian scientists were interested in ageing since the nineteenth century and this continued after the revolution.^17^ Luminaries of earlier Soviet ageing research included Aleksandr Bogomolets and Aleksandr Nagornyi who both worked on gerontology in Ukraine in the late 1930s, with Bogomolets holding a conference on the subject in 1938. Ukraine and Kyiv again became the site of Soviet gerontological research after the Second World War when the Institute of Gerontology and Pathology AMN USSR (affiliated to the Soviet Union’s Academy of Medical Sciences) opened there in May 1958.^18^ Part of the rationale for the later Soviet interest in gerontology was demographics: the gerontologist Konstantin Duplenko wrote in 1985 that the number of persons aged over 60 increased from 13 million in 1939 to 21 million by 1959.^19^ Keeping older people healthy and working for longer consequently formed part of the research programme on ageing. Researchers working in the Kyiv Institute or affiliated to it in some way produced much of the archival and published sources in this article.

In the Soviet case, many of those interested in gerohygiene in the 1960s came from a social gerontology background. Social gerontology had been established as a new branch of gerontology in the USA and Western Europe after the Second World War and connected to the medical-biological as well as the ‘economic, political, demographic, and psychological’ aspects of gerontology.^20^ Social gerontology did not arrive in the USSR until later, but researchers there were quick to support the field and it came under the remit of the Institute of Gerontology’s hygiene or gerohygiene section (it had three sections, the others being experimental and clinical).^21^ One scholar writing in the 1970s noted that, although social gerontology as a discipline in the USSR came later than in the West, it was already present in the work of scientists I. I. Mechnikov (Elie Metchnikoff) and Bogomolets and that hygienists and geriatricians, not sociologists, developed the field.^22^

Soviet social gerontologists defined gerohygiene as ‘designed to study and solve complex problems in industrial hygiene disciplines in relation to older age populations’ and related to a range of disciplines including economics, demography and psychology.^23^ The Soviet interpretation of gerohygiene was multi-disciplinary, attracting a range of scientists, scholars and practitioners. It also had important practical application. The Soviets were cognizant of the fact that ‘it is often less costly to prevent disease than to treat it’.^24^ A prematurely ageing population had serious economic consequences that could cost billions of rubles per year.^25^ Gerohygiene could help prevent premature ageing and improve people’s working capacity.

The connection between premature ageing and the ability of older people to work meant that gerohygiene was ‘firmly in the practice of public health’.^26^ The Soviet Ministry of Health and the presidium of the Academy of Sciences endorsed research on gerohygiene and both agreed that there was a need for ‘further expansion of research on social hygiene questions and other directions of gerohygiene’.^27^ The results of the research were to facilitate ‘scientifically based recommendations in the areas of labour and rest, diet, and the behaviour of the older age population’. Social hygiene for older adults was also to be integrated into textbooks for medical educational institutes and for doctors taking special courses in geriatrics at the Kyiv Institute for the Advanced Training of Doctors.^28^ In this way, some of the research being undertaken on gerohygiene made its way from theory to practice.

Chebotarev considered gerohygiene to have ‘huge practical significance in the search for methods to prevent premature ageing’ and looked beyond Soviet borders.^29^ As an integral part of the wider scientific and medical study of ageing, Chebotarev and his colleagues wanted researchers abroad to buy into gerohygiene. When Soviet researchers took to the world stage at the International Congress of Gerontology in Kyiv in 1972, they championed gerohygiene. Noting the importance of social issues for ageing societies, delegate Siegfried Eitner observed: ‘This point of view was strongly emphasised at the 9th International Congress of Gerontology in Kyiv, where gerohygiene was promoted as an indispensable research field of gerontology and as necessary for modern society’.^30^

The Soviet Union was not alone in its interest in gerohygiene.^31^ Siegfried Eitner and his colleagues in East Germany developed gerohygiene research and in the West gerohygiene ideas existed in the form of industrial gerontology.^32^ Eitner, Chair of Occupational Therapy at Berlin’s Humboldt University, defined gerohygiene as ‘part of hygiene and gerontology’.^33^ Although he noted that the Soviet Union, Romania and other socialist countries were already active in planning around old age and health,^34^ when it came to ageing issues in the 1950s and 1960s, Eitner and his colleagues turned to West Germany, rather than the East.^35^ The 1970s saw more collaboration with the Soviet Union, however. Knowing a great deal about the Institute of Gerontology in Kyiv, Eitner wrote in 1975 that there was ‘much to learn’ from the Institute’s ‘exemplary’ work and wider Soviet approach to ageing issues.^36^ Commenting on the links between research and practice, Eitner noted:

Social gerontology and gerohygiene prove to be promising branches of ageing research in socialist society. Without their participation in gerontological research, the questions of gerontology and modern medicine cannot be solved, a thesis that Professor Chebotarev pointed out at the 9th International Congress in Kyiv in 1972 and again in Berlin (at the 4th Congress for the Society of Ageing Research in the DDR) in 1973. The fact that fundamental research must be intensified within the framework of large-scale research and socialist as well as international cooperation is not a contradiction to the above-mentioned thesis. The solution to the ageing problem lies in the well-balanced symbiosis of biological, medical, sociological, and psychological ageing research.^37^

Eitner and Chebotarev were preaching from the same hymn sheet when it came to gerohygiene. Although East Germany had a different ageing demographic to the Soviet Union, as well as its own history of gerontological research, ideas of gerohygiene appealed to researchers in the two socialist states. The mid-1970s saw bilateral agreements on scientific cooperation drawn up between the USSR and GDR. These included collaborative research between institutes on the theme of ‘Principles and methods for preparing for active ageing and increasing the level of social adaptation in retirement’ and a bilateral agreement between the Institute of Gerontology AMN USSR and the Ministry of Health GDR for a research project on human bone tissue and the ageing process.^38^ Eitner also collaborated with colleagues from the Institute of Gerontology. In a co-authored paper with E. Stezhenskaia and N. N. Sachuk, Eitner discussed a 5-year plan for cooperation in social gerontology and gerohygiene that focused on themes in preparing for active old age. In this plan, they paid particular attention to old age workers, preparation for a ‘rational’ retirement and dispensarisation^39^ in industry for older workers.^40^ Gerohygiene thus proved fertile ground for socialist scientific discussion and collaboration.

The concept of gerohygiene in many ways presaged contemporary discussion of how to define old age and to view the ageing process as one shaped by a range of factors other than biology or chronological age.^41^ Indeed, ideas around gerohygiene reflected more recent definitions of ‘active ageing’, although the socialist context meant that healthy ageing in the Soviet Union was geared towards the end goals of collective well-being and productivity rather than individual happiness per se. In 2002, the World Health Organization defined active ageing as ‘the process of optimizing opportunities for health, participation and security in order to enhance quality of life as people age’.^42^ Similarly, one of several objectives at the Second World Assembly on Ageing, in Madrid, 2002, was ‘recognition of the social, cultural, economic and political contribution of older persons’.^43^ These concepts around healthy and active ageing and a rounded approach to elder health were not far removed from the research agenda of the Institute of Gerontology in Kyiv, or the socialist world more generally. Researchers at the Institute covered many themes in their work ranging from preventing premature ageing to infectious diseases and older people.^44^ Those at the Institute of Gerontology examined gerontology and geriatrics holistically, with laboratories and departments focusing on biological, clinical and social gerontology and geriatrics.^45^

Concepts around active ageing meshed with Khrushchev’s push for greater collectivism in Soviet society and the broader socialist goal of making people into active members of the community. Ageing actively spoke to the generation of the 1920s and 1930s, many of whom had built the revolution and did not intend to cease their political, social and cultural contributions just because they had reached a certain age.^46^ Elder participation in the collective was also a feature of the Brezhnev government, when Second World War veterans, and from the late 1970s labour veterans, were held in particularly high regard. This continued into the early 1980s after Brezhnev’s death. ‘It is necessary’, claimed Yuri Andropov at a meeting of Communist party veterans in 1983, ‘to increase the role of veterans in all spheres, and…to have them more involved in economic and social life’.^47^

Although welfare reforms and improving living standards were on the agenda in the late socialist period, Cold War politics and the exertions of building socialism took their toll on people’s health. State spending prioritised the military-defence economy. Although science was valued and promoted during these years, ordinary Soviet citizens often lacked access to good quality healthcare and medicine. Mark G. Field has written about a gradual ‘health crisis’ after the mid-1960s that ‘affected the entire population, from infants to the elderly’.^48^ Field attributes the long crisis to increased defence spending and reduced healthcare funding under Brezhnev, corruption, paternalism and rising infant mortality. Older people’s health also suffered owing to conditions of civil war, famine, the Second World War, and related privations and/or an unhealthy lifestyle (alcoholism, smoking, difficult working conditions).^49^

But Soviet gerontologists fought against the negative trend in healthcare outcomes. While an editorial in *The Lancet* in 1999 claimed about the USSR that: ‘Preventative medicine consisted of screening check-ups rather than population-based health-promotion measures’, the work of researchers based at the Kyiv institute shows that health promotion for older adults was a crucial part of their work.^50^ Promotional work could address some of the ‘health crisis’ deficiencies. A 1984 WHO study showed that a person’s health is ‘only 10% a function of health services’, a trend also noted in Soviet research.^51^ Gerohygiene, with its focus on the broader environment, had a bearing on a person’s health and, at least in terms of physical activity, offered the individual some control over the ageing process and their general well-being.

The Soviet state expected middle-aged and older citizens who were still fit for work to continue being socially engaged and to help the economy. By the 1980s, the nature of gerohygiene work included ‘improving older age workers’ general and professional working ability and social activities, mental and physical wellbeing, and maximizing the contribution of older age workers in the economic interests of the country’.^52^ The state called on its greying generations to continue their personal transformation and to partake in economic, social and cultural life. By preserving their health, they could still play an important part in constructing communism. Although the wider healthcare infrastructure and living conditions were often not conducive to achieving this, aspects of gerohygiene nonetheless presented older people with the opportunities to age actively and participate in socialist life.

## Getting active: physical activity and elder health

While active ageing speaks to more than just physical activity, exercise is crucial in maintaining good health in older age. As scholars have recently noted: ‘Among the behavioral and lifestyle factors, PA [physical activity] is the most important determinant of “active ageing” and has a major role in improving the quality of life, in reducing disability, and in the “compression of morbidity” in later life’.^53^ Physical activity is known to improve mental health, reduce the likelihood of chronic diseases and have a range of economic and social benefits.^54^ Research examining the impact of physical activity on a person’s health and their ability to work featured regularly in the Soviet literature on gerontology.^55^ Soviet researchers were keenly aware of the benefits of exercise for maintaining health in old age and warding off premature ageing.

Gerontologists concluded that certain types of physical exercises were necessary for older people and that physical culture was ‘one of the most important tasks of scientific research’ on the ageing physical body.^56^ It was ‘[e]specially significant in relation to the physiological peculiarities of the ageing individual’, wrote one expert on physical culture.^57^ Research on physical culture in the 1960s was so pervasive that it featured widely in conferences on gerontology and geriatrics, a development that, according to I. V. Muravov – an author of several books on the subject, and a regular on the lecture and television circuit – called a testament to the ‘huge significance of physical culture in achieving longevity’.^58^ He advocated making physical culture a part of people’s lives before they reached age sixty.^59^

The 1960s was a particularly vibrant period for research on physical culture and ageing. In 1963, a conference in Kyiv was dedicated to the topic of motor activity (*dvigatel’naia rezhima*) and ageing with an edited volume based on proceedings published a couple of years later.^60^ As the editors noted, ‘only close contact and a creative interrelationship between gerontology and physical culture can have the most effective pedagogical, psychological, physiological, and medical justification for the use of physical culture for middle and elderly people, developed and introduced to the practice of rational forms of organised activity for a person in their mature years and old age’.^61^ Acting on the basis of research in the area, the Institute of Gerontology often made recommendations, such as introducing physical culture exercises into medical institutions.^62^ Its scientific council, for example, concluded that research on sport and gymnastics could be developed as a guidebook for middle-aged and older people to practice specific exercises themselves.^63^ Much of the research was required to have elements of practical implementation, and so older people in the local community became an integral part of its work. These efforts to apply research to practice fitted in with the late Soviet state’s ideological and political programme, which encouraged its citizens to be activists, mobilise the collective and make themselves and the community at large into better socialists.

Scientific and medical research were primarily to help people to achieve ‘normal longevity’,^64^ which I take to mean average life expectancy rather than the kind of record-breaking ages achieved by long-livers. In the early 1960s, the average life expectancy was 68 years.^65^ Soviet scientists conducted extensive research on exercise and physical activity for middle-aged and older people, targeting the impact of certain types of exercise on an older person’s organs, circulation, respiratory system, cardiovascular health and muscles.^66^ They then published and disseminated their research findings – again with the aim of not just advancing the field of gerontology but informing the public on how to become healthier. Their findings also became part of the expanding field of geriatrics, gradually informing medical practice.

The Soviet Academy of Medical Sciences and the Ministry of Public Health, for example, were interested in ways to ‘increase longevity and provide for an active old age’.^67^ Physical culture presented a particularly collaborative approach to tackling this goal of making physical activity in old age accessible and practical. In October 1960, the Academy of Medical Sciences, the Committee for Physical Culture and Sport (under the Council of Ministers) and the Lesgaft Institute of Physical Culture in Leningrad corresponded with one another regarding the Academy of Medical Sciences’ programme for practical events in the sphere of gerontology.^68^ In this correspondence, Professor V. M. Dobrovol’skii, head of the sports medicine faculty at the Lesgaft Institute, outlined four key areas in gerontology in which they could become involved.^69^ The first involved providing support to physical culture exercises conducted with older people from the Gorky Leningrad home for scientists. The second was to conduct seminars for doctors (planned by the Institute of Gerontology). Third on the list was publishing articles about physical culture and older people in the institute’s yearbook *Gerontology and Geriatrics* (*Gerontolog iia i geriatriia*) and an additional brochure on themes such as ‘gymnastics in old age’. Finally, the faculty would also produce popular lectures on physical culture in old age.^70^ This multi-pronged approach reflected a clear interest in coordinated research and publications on physical culture for older people. Around this time, researchers from the Institute of Gerontology, the Central Scientific-Research Institute of Physical Culture in Moscow, the Leningrad Institute for the Advanced Training of Doctors and the Moscow Province Pedagogical Institute were also involved research on ‘active old age’ and the links between physical activity and ageing.^71^ Collaboration and a quest to inform and educate older people characterised the late Soviet approach to ageing.

In the Institute of Gerontology’s laboratory for functional diagnostics and physical culture, scientists conducted research that could be used in medical practice so that doctors would be able to help greater numbers of older people include specialist exercises (specifically designed for their health condition, age, fitness level and gender) in their daily routines.^72^ The head of the geriatric office of Sochi’s central polyclinic, for example, reported that the presence of an instructor and doctor of therapeutic physical culture affiliated to the local city physical culture dispensary (*gorfizkultdispensar*) and a doctor from the Moscow Institute of Physical Culture was a great help to groups of older people practicing therapeutic physical culture and that older people followed exercises on the television.^73^ Laboratory research translated to the dispensary and onto the television to provide older people with access to physical culture. For a generation familiar with morning *zariadki* (callisthenic style exercises) on the radio, this kind of access allowed older people to exercise safely and take charge of their health.

Some of this research expanded and diversified to reach older people across the Soviet Union through a variety of channels. Elements of Muravov’s 1960s work included ‘developing gymnastics, “health groups,” and “health zones” in Ukraine and the USSR’.^74^ He pressed for ‘health groups’ to be flexible, organised on a broader level than just sports centres.^75^ (Local medical and physical culture dispensaries oversaw health group activities.)^76^ He wanted to see housing authorities involved in ‘health groups’ and a greater move towards older people being able to engage in physical culture independently. Muravov completed this research in December 1964 and found that exercises for middle-aged and older people, when carried out systematically in ‘health groups’, improved their physical ability and recovery.^77^ Not only did these ‘health groups’ improve older people’s health, but they also fitted in well with socialist ideas around collective organisation and mobilisation.

Physical training conducted in ‘health groups’ in the late 1960s was shown to improve circulatory, respiratory and muscular function in older people (based on a sample of 40 people aged 60–69).^78^ This kind of research was put into action. Z. G. Revutskaia noted that physical training in health groups proceeded ‘according to special physiologically grounded programmes’ and that by the mid-1970s they were a ‘mass physico-cultural keep-fit institution for the aged to which patients are recommended by district physicians’.^79^ The ‘grey’ generation would already have been acquainted with similar kinds of keep fit programmes participating in or attending physical culture parades or the GTO (*Gotov k trudu i oborone*/Get Ready for Labour and Defence^80^) events. This was a playbook already used to mobilise youth after the 1917 revolution. Now, in their old age, pensioners were asked to remain committed to exercise for the good of their health and to help bolster the Soviet economy through their continued labour. This point was emphasised in the preface to the Russian translation of Australian Dr. Russell Gibbs’ 1981 book on exercises for the over fifties (*Esli vam za 50*).^81^ Commenting on the book, A. P. Laptev notes that citizens of all ages in the Soviet Union, unlike in the West, could participate in an organised state sports system from an early age and older people could join physical culture collectives, health rooms and other outlets for physical activity.^82^

Soviet scientists advanced research on physical culture and ageing before their counterparts in the West spilled ink on the topic. Indeed, J. C. Brocklehurst, the editor of a comparative book on geriatrics published in 1975 (and later one of the UK’s leading geriatricians) commented that in the case of the USSR ‘the particular emphasis on physical culture…is probably less typical of other countries’.^83^ Of all the countries represented in the volume (Great Britain, the USA, the Netherlands, Sweden and Australia), the USSR was alone in discussing physical culture and exercise as part of a prophylactic campaign to promote better health among older people. As Revutskaia wrote in the volume, prophylactic and therapeutic approaches to medicine, physical culture and sanitary measures were ‘designed to allow older people wherever possible to remain in their normal environment, to prolong their working period and to preserve mobility and the ability for self-service’.^84^ This latter point on ‘self-service’ is vital for older people if they are to remain independent. In a world that categorised all older people as ‘vulnerable’ and asked them to ‘cocoon’ to save themselves from the 2020–21 Covid-19 pandemic, we would do well to remember the agency and independence of older people.

## Labour, active rest and the older person

At an Institute of Gerontology meeting in 1961 a certain Professor Rubakin from Moscow asked: ‘We have 20 million pensioners, who of these cannot work?’^85^ In 1970, there were 36.2 million people of pension age, of whom 4.5 million, or 12.4%, worked.^86^ By 1976, the number of those older than the ‘official working capacity age’ was 39.4 million, or 15.4% of the population.^87^ The number of older people was expected to rise to 20 million, or 17%–18% of the population, by 1979.^88^ As historians such as Lovell, Klots and Romashova have argued, older people represented significant economic capital, and indeed, ‘moral capital’. Older people, often highly qualified, could ‘make up for the deficiencies in state services’.^89^ Looking after elder health was consequently beneficial to both pensioners and the economy.

Even though the official retirement age was 55 for women and 60 for men, the Soviet government supported those who wanted to remain in paid employment. On 31 December 1969, the Soviet Council of Ministers passed a resolution ‘On measures to further increase the material interests of able-bodied old age pensioners in continuing to work after the commencement of a pension’.^90^ That year the Soviet Council of Ministers had also issued resolution No. 705 from August 1969 ‘On measures for expanding the use of pensioners, invalids, and persons in the household in industries to produce consumer goods and services’. This was followed by further legislation on older people in the workplace in the 1970s, including resolution No. 674 from September 1973 ‘On measures for the further development of better use of labour of pensioners by age and disability in the national economy and additional benefits’ and resolution No. 850 from September 1979 ‘On measures to materially stimulate work by pensioners in the national economy’. ^91^ These resolutions looked at ways to accommodate and broaden the working potential of older people and improve conditions for pensioners working in specialised industries. They showed that the Soviet government was, by the 1970s, keen to incorporate older people in the workforce.

Soviet state interest in finding ways to accommodate pensioners in a range of economic and social activities presented researchers with a mandate to investigate ways to preserve elder health and assess their ability to work. Scientists researching physical activity for older people could see how forms of exercise could be harnessed to address some of the issues around not just elder health and premature ageing but also working capacity. One of the ways of alleviating the burden of physical and mental exertion was active rest or active leisure (*aktivnyi otdykh*), which could be applied to both intellectual and manual labour. I. M. Sarkizov-Serazini, from the faculty of medical control and therapeutic physical culture at the Central Institute of Physical Culture, espoused active rest in the form of walking, tennis, swimming and other forms of exercise for an hour to an hour-and-a half each day as a means of preventing premature ageing.^92^ ‘Spending time in the fresh air with a reasonable amount of physical exertion’, he surmised, would ‘increase general working ability’.^93^ By engaging in work and physical exercise older people would enjoy ‘positive emotions’, helping them to train and strengthen their bodies, but they would also contribute to labour.^94^ Sport and regular exercise, Sarkizov-Serazini emphasised, was not just for the young and was vitally important in protecting against illnesses typically associated with old age as well as a way of prolonging youth.^95^

Realising that insufficient muscular development could contribute to premature ageing, Muravov also extolled the benefits of physical culture and underlined its importance for both industry and the individual.^96^ Physical culture was a key component in the Soviet effort to improve older people’s health and Muravov’s work on active rest showed that post-work changes or exertions in the circulatory and respiratory system could be restored quickly after ‘rational forms of active rest’.^97^ Physical activity could preserve the health of older people and help the Soviet economy. Researchers in Ukraine actively promoted their ideas around working ability and old age. They endeavoured to expand older age workers’ knowledge of physical culture through holding seminars and conferences on the subject.^98^ Physical culture and exercise were certainly holding their own in prophylactic efforts to prevent premature ageing and help people work for longer.

But Soviet ideas around active rest or active leisure were still in the process of being defined in relation to middle and older age people. The term active rest entailed any kind of activity that counterbalanced intellectual or manual labour. Various experiments and studies had demonstrated the benefits of active rest, as opposed to passive forms of rest. Originally developed by physiologist Ivan Sechenov in 1903 and known as the ‘Sechenov phenomenon’, active rest was based on the relationship between the body and labour, examining the increased exhaustion levels of a person’s arms after a period of work.^99^ The contemporaneous understanding of active rest, as outlined in a lecture by the Institute of Gerontology’s S. A. Tanin, was that it developed the process of slowing down the central nervous system’s fatigued muscles, thus ‘hastening recovery and leading to increased working ability’.^100^ Tanin provided several examples to illustrate this point. The well-known Russian author Lev Tolstoy, he explained, recovered from his literary endeavours by working in the fields, while the famous Russian scientist Ivan Pavlov played *gorodki* and tended his vegetable plot as a form of ‘active rest’.^101^ Scientists figured that research on active rest could be transposed to industry where repetitive motion took its toll on the body. It is hardly surprising then that research became increasingly connected to occupation, where older people’s health could be examined in relation to their professional environment.^102^

Even though Tanin referred to active rest and labour ability, these concepts were still works-in-progress. Sechenov’s work on active rest or active leisure had already inspired a range of research since the 1930s and Tanin’s findings were part of a broader field of research on the area.^103^ Still, Tanin actively disseminated his work: in 1961 he was penned to talk about ‘active rest and longevity’ on the radio and deliver a lecture on rest and labour for older people at the Arsenal factory in March 1960. He also delivered a talk on active rest to an audience of seventy at a home for elderly people in April of that year.^104^ Promoting their research findings to academic and public audiences was central to the work of those at the Institute. These dissemination activities were part of a longstanding ‘enlightenment’ effort to inform people, giving them the tools to make themselves healthier and become ‘better’ Soviet citizens.

Although a defined retirement age existed in the Soviet Union, there was no age limit to prevent people from contributing to the communist cause. ‘The Soviet government’ in hygienist Sarkizov-Serazini’s words, ‘valued the work of the older generation’. A form of social contract existed between the state and its older citizens when it came to healthcare and labour.^105^ Older people, like the youth, were to be ‘active builders of the new life’.^106^ Government resolutions on labour and welfare for older people made it clear that they envisaged older people working beyond retirement age. These kinds of policies and resolutions were not entirely out of line with what older people wanted.

Soviet studies and surveys indicated that retirement was not wholly driven from the top down and discussions on the issue suggested that pensioners wanted to remain in employment for financial reasons but also to maintain their routine.^107^ In the Soviet context, where so much importance was placed on work and the construction of communism, people’s identity, status and way of life were closely connected to their place of work. The close tethering of social and cultural life to the workplace collective meant that retirement consequently presented a particular challenge.^108^ In this context, the research of gerontologists makes sense. Much of their work focused on exploring ways to help older people not just live longer but also work for longer. Even if the studies and surveys were designed to confirm Soviet policies, their findings tally with attitudes of older people current in modern societies. The WHO’s World report on ageing and health cites surveys from the USA showing that ‘the majority of people approaching traditional retirement age do not actually want to retire’ due to poverty, but also owing to ‘broader interest in remaining active participants in society’.^109^ In the Soviet Union, a whole section of gerohygiene was devoted to labour and the ‘organisation of forms of social and occupational activity’.^110^ The issue of older people in the workplace occupied Soviet gerontologists (and gerontologists in Western Europe and the USA) from the 1940s and 1950s. This is an issue that requires a sensitive understanding of a population group that is defined by age. For that reason, it is worth pausing here to examine some specific terms and concepts.

Soviet gerontologists discussed and defined the terms they used. Working ability (*trudosposobnost*’), which can also be translated as working capacity, was a word gerontologists used frequently. Books were published with this word in the title. Work therapy, in Russian ‘*trudoterapiia*’ or ‘*trudovaia terapiia*’, was closely connected to ideas around working capacity, health and age. One scientist concerned with defining some of the common terms used by her colleagues brought this word to their attention at a plenary meeting of the institute’s Problem Commission and Scientific Council in December 1961. Therapist and cardiologist, Professor Maria Iosifovna Khvilivitskaia, said that working ability was ‘very important’ and ‘reflects the condition of the organism as a whole’.^111^ But before that, she wondered about frequently used terms such as ‘elderly’ (*pozhiloi*), ‘aged’ (*prestarelyi*) and ‘older group’ (*starcheskaia gruppa*). ‘What age do we understand elderly to be, and what age do we understand aged to be?’ she asked.^112^

These definitions were problematic. As Khvilivitskaia, who was based at the Leningrad Institute of Expertise for Working Capacity and Organisation of Work for Disabled People, pointed out, taking the 65–75 age group as ‘early old age’ and the age 75 and older as ‘late old age’ was ‘absolutely arbitrary’ and ‘without any scientific basis’.^113^ Moreover, she recognised that this understanding of elderhood was at odds with the official retirement age in the Soviet Union.^114^ She threw another spanner in the works: broaching the topic of ‘premature ageing’ (*prezhdevremennaia starost’*), she wondered about ‘timely ageing’ (“*svoevremennaia starost’*). In clinical work and in research about labour capacity she argued that a person’s ‘passport information’ was not everything; instead, Khvilivitskaia urged that a person’s ‘level of senescence’ also be factored into research.^115^ These questions touched on issues about age and retirement, issues that were global in nature.

Soviet scholars were familiar with wider international debates about raising and lowering retirement age, as well as general issues around pensioner health. Some Soviet scholars noted that lectures on themes about health, retirement and leisure for older people were given in Glasgow since 1959, while others noted that ‘the US, England, West Germany, and Scandinavian countries were discussing increasing the pension age to 68–70’.^116^ While issues around age, retirement and health were not specifically Soviet, class and ideology characterised Soviet discussions. Older people in the USSR could retire earlier to protect their health but Soviet scholars argued that this option was not available to people in the West.^117^ Comparing the socialist and capitalist systems in 1969, Soviet scholars invariably concluded that their citizens fared better. Khvilivitskaia and her colleague Kalinina followed this narrative when they wrote: ‘The Soviet state, by providing old-age pensioners with the opportunity to work without any harm, and in most cases with health benefits, protects the interests of these persons and at the same time expands its labour resources’.^118^ But this narrative had begun to fall apart in the mid-1980s, perhaps a consequence of Mikhail Gorbachev’s *glasnost* (openness) policy. By then Soviet researchers recognised that both systems struggled to protect workers against the demands of modern industry.^119^

Narratives aside, Khvilivitskaia’s research on older people and work raised several pertinent questions about the parameters of old age and working ability. At the massive Kirov factory, she noted, there were workers aged 80 years and older who were ‘extremely valued in the factory’.^120^ The factory had found a way to accommodate these older workers, she noted, but she called for greater scientific research into the kind of conditions that would allow older people to work.^121^ In the workers’ state, prioritising occupational safety and well-being was a key objective (though one that often remained in the realm of rhetoric than reality).

Conversations such as those initiated by Khvilivitskaia underscored the engaged nature of scientific research on gerontology; it was an area of intense debate and one that was still relatively new in the Soviet Union. Similar discussions were underway in the USA where there was ‘a body of practitioners charged with the task of facilitating the training and employment of the so-called older worker’ and challenging understandings around work and retirement.^122^ Eitner and his colleagues in East Germany (where 85.5% of men and 28.5% of women worked beyond retirement age)^123^ were also active on this front. In the 1970s, Eitner analysed the gender-related behaviours of older people at retirement age, the advantages and disadvantages of working past retirement age, as well as issues around occupational health and hygiene for older people.^124^ Concerns about older people and the economy were also raised in the American Council of Ageing’s first Annual Report in 1963.^125^ British experts had already been discussing some of these issues in the late 1940s and 1950s, exemplified by the Nuffield Foundation’s research on the role of older people in industry.^126^

But instituting these research findings and recommendations was another matter. Despite the British Labour and then Conservative government’s efforts to help older people remain in work in the 1940s and 1950s, employers preferred to draw on young immigrant workers.^127^ Both US and Soviet researchers struggled to accommodate older people’s needs at work. In the USA, researchers and practitioners were confronted with the question of how to marry theory and practice in relation to ‘the older worker in American society’.^128^ Writing after the end of the USSR in the early 1990s, A. L. Retushiuk, who specialised in the physiology of labour at the Institute of Gerontology in Kyiv, was critical of Soviet and international lack of action on measures taken to help older people adapt to labour. He wrote that the ‘industrial gerontology’ research of the 1970s ‘never approached the problems of gerontological hygiene, physiology, and labour psychology’.^129^ One of the key issues for Reshetiuk was management, specifically ‘technocratic management’; he wanted to see a ‘humanization of technology and management’.^130^ Rather than introducing new technologies that enabled middle-aged and older persons to continue their work in industry, factory managers instead favoured using existing technology. ‘The biosocial conflict between the person and technology’. Reshetiuk wrote, was ‘often obviated by the elimination of the ageing person from work’.^131^

These early post-Soviet discussions presented a stark contrast to the idealism of Khvilivitskaia and others working in the late socialist years. Rather than promoting and accommodating older people, pensioners had now been effectively eliminated from the workforce. The relative stability of the welfare system constructed under Khrushchev and Brezhnev had crumbled, leaving older people exposed to the vagaries of the post-Soviet state. Reshetiuk’s calls for a gerontological humanisation of technology more likely spoke to a Soviet past more than the immediate post-Soviet present. But these efforts of scientists (Soviet or otherwise) to instigate change to fundamentally improve the health and well-being of older people were important. And questions about retirement age, defining old age and the contribution of older workers to the economy have not been resolved. With governments in Russia, Western Europe and the USA still debating these issues, we seem no closer to a resolution.^132^

## Conclusion

Researchers in the Soviet Union devoted considerable time to examining older people and gerohygiene. Much of the work in the area emphasised the role of physical activity and connected these to the overall importance of physical culture to health. While Europe was primarily interested in gerontology because of ‘falling fertility and family structures’, and the USA ‘the low labor participation rate’, the Soviet Union, though also concerned with these issues, was also concerned with shaping its citizenry and the collective pursuit of building communism. Soviet gerontological research was accordingly diverse and an ongoing response to scientific, political, economic and societal concerns.^133^ Soviet gerontologists were also keenly attuned to developments in other countries: they received English language training, attended international conferences and read foreign literature. The Institute even held its own conference in English because, as Chebotarev stated: ‘Mastering the English language is the duty of every scientific worker’.^134^ The world might not have been wholly aware of research on ageing in the USSR, but Soviet gerontologists were certainly up to date on international developments in their field.

The postwar period in the Soviet Union witnessed a growing recognition of the need to develop physical culture for older people. Scientists realised that special medical attention needed to be paid to elder health, especially if older people were expected to remain in the labour force into and beyond their fifties and sixties. Soviet interest in physical culture and gerohygiene for middle-aged and older adults also chimed with the ideological and political objectives of mobilising society around collectivist ideals and values. Being physically fit and healthy was important because it allowed people to engage with Soviet life and continue working. By following exercises on a television or becoming involved in physical culture classes, older people could preserve their health and in so doing contribute to Soviet society (if they chose to buy into this). The scope and intensity of research on gerohygiene, especially in relation to physical activity and labour, leaves us with an incredibly rich literature to draw on. Rather than reinventing the wheel when it comes to ageing research, we might do well by turning to Soviet (and indeed, socialist) gerontology and its holistic and expansive approach to growing old, especially its emphasis on prophylaxis.

## Footnotes

1 N.K. Witte *et al*., *Obraz zhizn’ i starenie cheloveka* (material sympoziuma), (Kyiv, Zdorov’ia, 1966), 3. Introduction by Chebotarev. I have translated *dzigatel’naia rezhima* as movement regime, but technically it translates as ‘motor regime’. Chebotarev was head of the Institute of Gerontology from 1961 to 1988. For more on Chebotarev and understandings of gerohygiene, see D.F. Chebotarev, ‘Razvitie Gerontologii *v* SSSR’, in *Aktual’nye problem gerontologii i geriatrii*, *Materialy ob’edinennogo plenuma pravlenii Vsesoiuznogo nauchnogo obshchestva patologoanatomov, Vsesoiznogo nauchnogo obshchestva gerontologov i geriatrov*, 16–18 January (Kyiv, 1968), 10; TsDAVO (Central State Archive of the Supreme Organs and Administrations of Ukraine), f. 4783, op. 1, d. 52, l. 1, 1961–62.

2 On subjectivities, see especially Jochen Hellbeck , Revolution on my Mind: Writing a Diary under Stalin (Harvard University Press, 2009). See also Nikolai Krementsov , Revolutionary Experiments: The Quest for Immortality in Bolshevik Science and Fiction (Oxford University Press, 2014). For subjectivities in the late Soviet period, see Anatoly Pinsky, Introduction (in English) to *Posle Stalina: pozdnesovetskaia sub’ektivnost’ 1953–85* (Saint Petersburg: European University St. Petersburg, 2018), available at https://www.academia.edu/35934285/Introduction_full_original_English_language_version_to_После_Сталина_позднесоветская_субъективность_1953_1985_СПб_ЕУСПб_2018_, accessed 3 May 2022.

3 For more on Soviet social policies see David L. Hoffmann, *Cultivating the Masses: Modern State Practices and Soviet Socialism, 1914–39* (Ithaca and London: Cornell University Press, 2011), Introduction, ch. 2.

4 See G.M. Ivanova, *Na poroge ‘gosudarstva vseobshchego blagosostoyaniia’ Sotsial’naia politika v USSR* (seredina 1950 kh – nachalo 1970 kh godov (Moscow: Institut Rossisskoi Istorii Ran, 2011). Thanks to Isaac McKean Scarborough for bringing this to my attention. See also Mark B. Smith, ‘The Withering Away of the Dander Society: The Pensions Reforms of 1956 and 1964 in the Soviet Union’, *Social Science History*, 39, 1 (2015), 129–48: 130.

5 For excellent work on youth, see Juliane Fürst , Stalin’s Last Generation: Soviet Post-War Youth and the Emergence of Mature Socialism (Oxford: Oxford University Press, 2012); Vladislav Zubok , Zhivago’s Children: The Last Russian Intelligentsia (Cambridge, MA: Harvard University Press, 2009), 34 . On the deterioration of older people’s health from the mid-1960s, see Mark. G. Field, ‘The Health and Demographic Crisis in Post-Soviet Russia: A Two-Phase Development’, in Mark G. Field and Judyth L. Twigg (ed.), *Russia’s Torn Safety Nets: Health and Welfare during the Transition* (New York: St. Martin’s Press, 2000), 24.

6 On gerontology in the UK and USA as analogous to the Soviet Union, see Isaac McKean Scarborough, ‘A New Science for an Old(er) Population: Soviet Gerontology and Geriatrics in International Comparative Perspective’, paper presented at a workshop in Liverpool 5 December 2019, 8–11.

7 For discussion on ageing and the West, see Kavita Sivaramakrishnan, *As the World Ages: Rethinking a Demographic Crisis* (Cambridge, MA: Harvard University Press, 2018), 92–6.

8

Atul Gawande , Being Mortal: Medicine and What Matters in the End (Metropolitan Books, 2014); Martha C. Nussbaum and Saul Levmore , Ageing Thoughtfully: Conversations about Retirement, Romance, Wrinkles, and Regret (New York: Oxford University Press, 2018).

9

James Chappel , ‘Old Volk: Ageing in 1950s Germany, East and West’, The Journal of Modern European History, 90 (2018): 792 .

10

Peter N. Stearns , Old Age in European Society: The Case of France (London: Croom Helm, 1977), 7.

11

Chris Phillipson , Capitalism and the Construction of Old Age (London: The Macmillan Press, 1982); Howard P. Chudacoff , How Old Are You? Age Consciousness in American Culture (Princeton: Princeton University Press, 1989); Thomas R. Cole , The Journey of Life. A Cultural History of Ageing in America (Cambridge: Cambridge University Press, 1992); Carole Haber and Brian Gratton , Old Age and the Search for Security: An American Social History (Bloomington: Indiana University Press, 1994); Haim Hazan , Old Age: Constructions and Deconstructions (Cambridge: Cambridge University Press, 1994); W. Andrew Achenbaum , Old Age in a New Land: The American Experience Since 1790 (Baltimore: The Johns Hopkins University Press, 2019 [originally published in 1978]). Chappel discusses many of these, see Chappel, ‘Old Volk: Ageing in 1950s Germany, East and West’, 792.

12 Stephen Lovell, ‘Soviet Russia’s Older Generations’, in Stephen Lovell (ed.), *Generations in Twentieth-Century Europe* (Basingstoke: Palgrave Macmillan, 2007), 219.

13

Lukas Mücke , Die allgemeine Altersrentenversorgung in der UdSSSR (Stuttgart: Franz Steiner Verlag, 2013).

14

Mark A. Edele , Soviet Veterans of the Second World War: A Popular Movement in an Authoritarian Society (Oxford: Oxford University Press, 2008); Edele, ‘“A Generation of Victors?”: Soviet Second World War Veterans from Demobilization to Organization, 1941–56’ (PhD Dissertation, University of Chicago, 2004).

15

Steven E. Harris , ‘“We Too Want to Live in Normal Apartments”: Soviet Mass Housing and the Marginalization of the Elderly Under Khrushchev and Brezhnev’, The Soviet and Post-Soviet Review, 32, 2–3 (2005), 143–74: 149.

16

Alissa Klots and Maria Romashova , ‘Lenin’s Cohort: The First Mass Generation of Soviet Pensioners and Public Activism in the Khrushchev Era’, Kritika: Explorations in Russian and Eurasian History, 19, 3 (2018), 597 .

17 For discussion of interest in ageing in the 1920s, see Krementsov, *Revolutionary Experiments*; Maria Tutorskaya, ‘Medical Propaganda: Miracles of Surgery and Fairytales’, in Susan Grant and Isaac McKean Scarborough (eds), *Geriatrics and Ageing in the Soviet Union: Medical, Social and Political* Contexts (London: Bloomsbury Academic, forthcoming, 2022).

18 For discussion of the Institute of Gerontology, see McKean Scarborough, *op. cit.* (note 6). For reasons of consistency and the fact that much of the source material is in Russian, I have used Russian rather than Ukrainian transliteration for the names of those working at the Institute of Gerontology (eg., Bogomolets rather than Bohomolets).

19 Duplenko, *Starenie. Ocherki razvitiya problem* (Leningrad: Nauka, 1985), 129. A violent civil war (1918–22), devastating famines (1920–21; 1933) and systematic state terror killed millions in the 1920s and 1930s. Some 27 million Soviet people died during Second World War.

20 M.D. Aleksandrovna, *Problemy sotsial’noi i psikhicheskoi gerontologii* (Leningradskii un-t: Leningrad, 1974), 9. Aleksandrovna traced the development of social gerontology in the USA, England and Denmark, drawing on the work of a host of international scholars, including Rosset, Tibbetts, Friis and Forssman.

21 D. F. Chebotarev, ‘25 let deiatel’nosti instituta gerontologii AMN SSSR *v* razvitii nauki *o* starenii’, *Vestnik Akademii Meditsinskikh Nauk SSSR*, 3 (1984), 304.

22 Aleksandrovna, *Problemy sotsial’noi i psikhicheskoi gerontologii*, 30, 40. Elie Metchnikoff was born in Kharkov (now Kharkiv, Ukraine) in the Russian Empire in 1845 and moved to Paris where he died in 1916.

23 ZG. Revutskaia, *et al.*, ‘Aktual’nye problem sotsial’noi gerontologii’, in DF. Chebotarev (ed.), *Gerontologiia i geriatriia 1969–70. Sotsial’naia sreda, obraz zhizni i starenie* (Kyiv, 1970), 3.

24
*Active Ageing: A Policy Framework*, World Health Organization. Noncommunicable Diseases and Mental Health Cluster. Noncommunicable Diseases Prevention and Health Promotion Department. Ageing and Life Course. A Contribution of the World Health Organization to the Second United Nations World Assembly on Ageing, Madrid, Spain, April 2002. Available at: http://apps.who.int/iris/bitstream/handle/10665/67215/WHO_NMH_NPH_02.8.pdf;jsessionid=E3FC7C1A3F7F9669EA019B311D1428F6?sequence=1, accessed 4 May 2021, 18.

25 TsDAVO, f. 4783, op. 1, d. 232, l. 62. 1969. Evdakov, VA. 1978 in Reshetiuk *et al.*, ‘Problemy profilaktiki serdechno-sosudistoi patologii *v* gerogigiene truda’, 75.

26 Aleksandrovna, *Problemy sotsial’noi i psikhicheskoi gerontologii*, 41; citing Witte, Stezhenskaia, 1969, 47. For more on the development of Soviet healthcare, see, for example, Susan Gross Solomon and John F. Hutchinson (eds), Health and Society in Revolutionary Russia (Bloomington: Indiana, 1990), Tricia Starks , The Body Soviet: Propaganda, Hygiene, and the Revolutionary State (Madison: The University of Wisconsin Press, 2008); Donald Filtzer , The Hazards of Urban Life in Late Stalinist Russia: Health, Hygiene, and Living Standards, 1943–53 (Cambridge: Cambridge University Press, 2010); Mark G. Field , Soviet Socialized Medicine (New York, London: The Free Press, Collier–Macmillan, 1967); Susan Grant , Soviet Nightingales: Care under Communism (Ithaca: Cornell University Press, 2022).

27 ZG. Revutskaia, *et al.*, ‘Aktualnye problem sotsial’noi gerontologii’, in *Gerontologiia i geriatriia. Sotsial’naia sreda, obraz zhizni i starenie* (Kyiv, 1970), 13.

28 Revutskaia *et al.*, ‘Aktualnye problem sotsial’noi gerontologii’, 13.

29 DF. Chebotarev, NB. Mankovskii, and VV. Frol’kis (eds), *Osnovy Gerontologii* (Moscow: Meditsina, 1969), 6.

30 Siegfried Eitner, Wolfgang Ruhland, and Helmut Siggelkow, *Praktische Gerohygiene. Handbuch der komplexen Betreuung im Alter* (Dresden: Verlag Theodor Steinkopff, 1975), 4.

31 For a brief overview of gerohygiene, see Siegfried Eitner, Wolfgang Ruhland, and Helmut Siggelkow, *Praktische Gerohygiene. Handbuch der komplexen Betreuung im Alter* (Dresden: Verlag Theodor Steinkopff, 1975), 17.

32 See *Bol’shaia Meditsinskaia Entsiklopedia*, entry by Stezhenskaia. Available at: https://бмэ.орг/index.php/ГЕРОГИГИЕНА, accessed 9 December 2019.

33 Siegreid Eitner *et al.*, *Praktische Gerohygiene*, 1. Soviet scholars defined occupational gerohygiene similarly, as ‘at the junction of gerontology and occupational hygiene’. AL. Reshetiuk, *et al.*,Problemy profilaktiki serdechno-sosudistoi patologii *v* gerogigiene truda‘, *Vestnik Akademii Meditsinskikh Nauk SSSR* 3 (1983), 70.

34 Siegried Eitner, *Gerohygiene hygiene des Alterns als Problem der Lebensgestaltung* (Berlin: Volk und Gesundheit, 1966), 26. The work of Ana Aslan in Romania is particularly well known in international gerontological circles. There is much research that could be done on socialist gerontology, but it is beyond the reemit of this article.

35

James Chappel , ‘On the Border of Old Age: An Entangled History of Eldercare in East Germany’, Central European History, 53 (2020), 363–4.

36 Eitner *et al.*, *Praktische Gerohygiene*, 15.

37
*Ibid.*, 393.

38 DF. Chebotarev and AV. Tokar,’ Nekotorye itogi *i* perspektivy nauchnykh issledeovanii, provodimykh *v* ramkakh Koordinatsionnogo Komiteta po issledovaniiam v oblasti gerontologii sotsialisticheskikh stran Evropy’, *Gerontologiia i geriatriia. Umstvennyi trud i aktivnoe dolgoletie.* Yearbook (Kyiv, 1976), 141. Cooperation extended to other Eastern and Central European countries.

39 In the 1970s, some of the earlier emphasis on labour productivity remained in the form of gerohygiene. Dispensaries, popular in the 1920s, regained visibility in the late Stalinist period. Chris Burton writes that the concept of dispensarization in the 1950s marked ‘an aggressive form of preventative health practices explicitly and largely devoted to raising labour productivity’. At this time, ‘dispensary observation was to be ranked by the occupation of the patient’. Chris Burton, ‘Medical Welfare During Late Stalinism. A Study of Doctors and the Soviet Healthcare System, 1945–53’, (PhD Dissertation, University of Chicago, 2000), 112.

40 Z. Aitner, N. Sachuk, and E. Stezhenskaia, ‘Problemy aktivnoi starosti’, in *Gerontologiia i geriatriia. Umstvennyi trud*, 142. For more on rational lifestyles, see Aleksandra Brokman, ‘“A Golden Autumn”: Mental Hygiene and Psychoprophylaxis of Old Age in Post-War USSR’, Working paper, 15–20.

41 For example, see Sivaramakrishnan’s argument, *As the World Ages*, 217.

42
*Active Ageing: A Policy Framework*, World Health Organization. Noncommunicable Diseases and Mental Health Cluster. Noncommunicable Diseases Prevention and Health Promotion Department. Ageing and Life Course. A Contribution of the World Health Organization to the Second United Nations World Assembly on Ageing, Madrid, Spain, April 2002. Available at: http://apps.who.int/iris/bitstream/handle/10665/67215/WHO_NMH_NPH_02.8.pdf;jsessionid=E3FC7C1A3F7F9669EA019B311D1428F6?sequence=1, accessed 4 May 2021, 12 (citation); 19.

43 Political Declaration and Madrid International Plan of Action on Ageing, Second World Assembly on Ageing, Madrid, Spain, 8–12 April 2002. Available at: https://www.un.org/esa/socdev/documents/ageing/MIPAA/political-declaration-en.pdf, accessed 4 May 2021.

44 TsDAVO, f. 4783, op. 1, d. 35, l. 15. November 1959. There were twenty-eight themes in 1959.

45 For more about how the institute came into existence, see McKean, *op. cit.* (note 6).

46 On grandmothers, see Danielle Leavitt-Quist, ‘The Modern Babushka: Rethinking Elderly Women after Stalin’, in Susan Grant and Isaac McKean Scarborough (ed.), *Geriatrics and Ageing in the Soviet Union: Medical, Political, and Social Contexts* (London: Bloomsbury Academic, forthcoming, 2022).

47 VD. Shapiro, *Sotsial’naia aktivnost’ pozhilykh liudei v SSSR* (Nauka, 1983), 4. Shapiro cites *Pravda*, 16, 20 August 1983.

48 Field, ‘The Health and Demographic Crisis in Post-Soviet Russia’, 24.

49 For good discussion, see Victoria A. Velkoff and Kevin Kinsella, ‘Russia’s Ageing Population’, in Field and Twigg (ed.), *Russia’s Torn Safety Nets*, 231–50, especially 233.

50 See ‘Health in Russia is broke, but who is to fix it?’ *The Lancet*, 353; 9150 (1999), 337. See also Field, ‘Health and Demographic Crisis’, 23.

51 The WHO (Fillenbaum) study showed that 50% depended on mode of life, 20% on environment and 20% on genetics. Cited in Vladislav V. Bezrukov, ‘Self-Care and Institutional/Non-Institutional Care of the Elderly’, in *Journal of Cross-Cultural Gerontology* 8 (1993), 351. Bezrukov wrote that the Soviet researchers noted similar findings in their own work.

52 Reshetiuk *et al.*, ‘Problemy profilaktiki serdechno-sosudistoi patologii *v* gerogigiene truda’, *Vestnik Akademii Meditsinskikh Nauk SSSR* 3 (1984), 75. On mental wellbeing and retirement, see Brokman, ‘“A Golden Autumn”’, 19.

53 Adrian Bauman, *et al.*, ‘Updating the Evidence for Physical Activity: Summative Reviews of the Epidemiological Evidence, Prevalence, and Interventions to Promote “Active Ageing”’, *The Gerontologist*, 56, S2 (2016), S268–80, here S269. Citing Crimmins (2015) and Kalache *et al,* (2002).

54
*Active Ageing: A Policy Framework*, World Health Organization. Noncommunicable Diseases and Mental Health Cluster. Noncommunicable Diseases Prevention and Health Promotion Department. Ageing and Life Course. A Contribution of the World Health Organization to the Second United Nations World Assembly on Ageing, Madrid, Spain, April 2002, 23. Available at: http://apps.who.int/iris/bitstream/handle/10665/67215/WHO_NMH_NPH_02.8.pdf;jsessionid=E3FC7C1A3F7F9669EA019B311D1428F6?sequence=1, accessed 4 May 2021 (WHO, 1998).

55 TsDAVO, f. 4783, op. 1, d. 37, l. 23.

56 Chebotarev and Stezhenskaia here referred to the 1964 symposium on ‘lifestyle and the ageing person’, TsDAVO, f. 4783, op. 1, d. 114, l. 64. Citation: TsDAVO, f. 4783, op. 1, d. 125, l. 43.

57 Motylianskaia *et al.*, ‘Dvigatel’nyi rezhim’, 172.

58 TsDAVO, f. 4783, op. 1, d. 51, l. 199. Protocol and stenographic record of the conference held at the Institute of Gerontology, Kyiv, 9–13 February 1961. On Muravov’s television and lecture appearances, see TsDAVO, f. 4783, op. 1, d. 52, ll. 73–4, 65.

59 TsDAVO, f. 4783, op. 1, d. 51, l. 201. He based his conclusion on papers presented at the 1961 Institute of Gerontology conference.

60 D.F. Chebotarev, *et al.*, *Fizicheskaia kul’tura – istochnik dolgoletiia* (Moscow: Fizkul’tura *i* sport, 1965), 3.

61 Chebotarev *et al.*, *Fizicheskaia kul’tura*, 4.

62 TsDAVO, f. 4783, op. 1, d. 37, l. 23.

63
*Ibid.*, 24. There is a wide literature on exercises for older people, see I. V. Muravov, *Fizicheskaia Kul’tura i aktivnyi otdykh v raznye vozrastnye periody*, Muravov, *Zdorov’e, trudosposobnost’ i fizichecskaia kul’tura* (Kyiv, ‘Znanie’, 1985); I.M. Tamarov, *Gimnastika dlia pozhilykh* (Medgiz, 1960); A.N. Startseva, *Fizicheskaia kul’tura zhenshchin pozhilogo vozrasta* (Medgiz, 1960); A.N. Trankvillitati, *Fizicheskaia kul’tura dlia zhenshchin v pozhilom vozraste* (Meditsina, 1964).

64 TsDAVO, f. 4783, op. 1, d. 31, l. 4. 1960.

65 TsDAVO, f. 4783, op. 1, d. 55, l. 78.

66 TsDAVO, f. 4783, op. 1, d. 52, l. 4.

67 TsDAVO, f. 4783, op. 1, d. 27, l. 70. 1960.

68 TsDAVO, f. 4783, op. 1, d. 45, l. 17.

69
*Ibid.*

70
*Ibid.*, ll. 17–8.

71 R.E. Motylianskaia, *et al.*, ‘Dvigatel’nyi rezhim *i* aktivnyi otdykh *v* profilaktike prezhdevremennogo stareniia’, in *Obraz zhizni*, 166–7. For other examples of wider cooperation, see I.T. Osipov, ‘Razvitiee nauchno-pedagogicheskogo napravleniia po ispol’zovaniiu fizicheskoi kul’tury v bor’be za dolgoletie cheloveka’, in Chebotarev *et al.*, *Fizicheskaia kul’tura*, 10–1.

72 TsDAVO, f. 4783, op. 1, d. 70, l. 91.

73 TsDAVO, f. 4783, op. 1, d. 60, l. 135. 1964.

74 TsDAVO, f. 4783, op. 1, d. 93, l. 14.

75 TsDAVO, f. 4783, op. 1, d. 94, l. 29; TsDAVO, f. 4783, op. 1, d. 115, l. 22.

76 T.N. Grosheva, ‘Meditsinskaia pomoshch’ naselaniiu starshikh. Vozrastnykh grupp v g. Voronezhe i meropriiatiia po ee uluchsheniiu’, (Dissertation, Voronezh, 1972), 22; citing N.S. Starodub, 1964.

77 TsDAVO, f. 4783, op. 1, d. 119, l. 26. Eitner recognised Muravov’s work, noting that he ‘was able to clarify control procedures in old age under the influence of physical exercises and active recovery’ (1963–9). Eitner *et al.*, *Praktische Gerohygiene*, 13.

78 TsDAVO, f. 4783, op. 1, d. 185, l. 34. O. V. Korkushko under the supervision of Muravov, 1967–8. A symposium on middle aged and older people’s health in resorts took place in Kislovodsk and Chebotarev hoped a similar conference might take place in Pyatigorsk in 1967. TsDAVO f. 4783, op. 1, d. 157, l. 61.

79 Z.G. Revutskaia, ‘The Union of the Soviet Socialist Republics’, in J.C. Brocklehurst (ed.), *Geriatric Care in Advanced Societies* (Lancaster: Blackburn Times Press, 1975), 133.

80 The GTO existed from 1931 and was for Soviet youth and adults between the ages of 10 and 60. It included a range of sports at different levels.

81 R. Gibbs, *Esli Vam za 50*, translation from English and introduction by AP. Laptev, 2nd edn (Moscow: Fizkul’tura *i* sport, 1985). Original published by Sun Books, Melbourne.

82 Laptev, in Gibbs, *Esli Vam za 50*, 2. Laptev notes that Gibbs points out this deficiency in the capitalist system.

83 J.C. Brocklehurst commenting on Doctor Revutskaia’s chapter in the introduction. J.C. Brocklehurst (ed.), *Geriatric Care in Advanced Societies*, 3.

84 Revutskaia, ‘The Union of the Soviet Socialist Republics’, 129.

85 TsDAVO, f. 4783, op. 1, d. 60, l. 27. This figure is like that projected by Duplenko, cited earlier (21 million people aged over 60 by 1959). The demographer Urlanis presents a considerably lower number for 1959, just 2 years earlier. At that time, there were 10.7 million people of retirement age (7.15% or 3.5 million were men and 7.2% or 7.2 million were women. B. Ts. Urlanis, *Dinamika i struktura naseleniia SSSR i SShA*, in N.K. Witte and E.I. Stezhenskaia, ‘Gigiena i fiziologiia truda naseleniia starshikh vozrastnykh grupp’, in *Osnovy Gerontologii*, 519. The 1959 census put the number of ‘old and elderly’ people at 12 million, or 12% of the population. TsDAVO, f. 4783, op. 1, d. 27, l. 70. Irrespective of statistics, it was clear that the number of older people in the Soviet Union was increasing.

86 L.M. Artem’eva, ‘Ekonomiko-statisticheskie aspekty zaniatnosti pensionerov po starosti’, in Belov (ed.), *Trudosposobnost‘pensionerov po starosti*, 141. Of this percentage, 3.9% of work was represented in the national economy.

87 B.P. Belov (ed.), *Trudosposobnost‘pensionerov po starosti, voprosy stimulirovaniia i organizatsii ikh truda* (Moscow, 1975), 5. Belov provides a good survey of research being conducted on older age people and industry.

88 TsDAVO, f. 4783, op. 1, d. 27, l. 70.

89 Lovell, ‘Soviet Russia’s Older Generations’, 219; citations in Klots and Romashova, “Lenin’s Cohort”, 581. Lovell is also cited in Klots and Romashova.

90 V.A. Acharkan, ‘Sotsial’no-pravovye mery, sposobstvyiushchie povysheniiu trudovoi aktivnosti pensionerov’, in Chebotarev (ed), *Gerontologiia i geriatriia 1969–1970. Sotsial’naia sreda, obraz zhizni i starenie*, (Kyiv, 1970), 29. A similar resolution was passed in November 1964.

91

A.A Dyskin and A.L. Reshetiuk , Zdorov’ye i trud v pozhilom vozraste (Leningrad: Meditsina, 1988), 144–6.

92 I.M. Sarkizov-Serazini (ed.), *Fizicheskaia kultura v pozhilom vozraste* (Moscow: Fizkul’tura i sport, 1956), 25.

93
*Ibid.*, 252.

94
*Ibid*, 7.

95
*Ibid.*, 3–4, 7. See also I.V. Muravov, ‘Fizicheskaia kultura i aktivnoe dolgoletie’, *Fizkultura i sport* 10 (1979), 82.

96 TsDAVO, f. 4783, op. 1, d. 55, l. 81.

97 TsDAVO, f. 4783, op. 1, d. 75, l. 55. 1962.

98 Sarkizov-Serazini, *op. cit.* (note 92), 7. At a republican wide seminar 550 workers participated, while conferences in Nikopol and Kherson drew 350 delegates.

99 TsDAVO, f. 4783, op. 1, d. 33, l. 184. I.M. Sechenov (1829–1905) was a leading Russian scientist who worked on neurophysiology and psychology. For an overview, see L.S. Guzman and E.G. Ianenko, ‘Effektivnost’ vliianiia razlichnykh form aktivnogo otdykha na rabotosposobnost’ *v* usloviiakh prodolzhitel’noi povtornoi deiatel’nosti’, in N.K. Witte, *et al., Obraz zhizni I starenie cheloveka (materialy simpoziuma)*, (Kyiv, “Zdorov’ia”, 1966).

100 TsDAVO, f. 4783, op. 1, d. 33, l. 186. For similar discussion, see I.V. Muravov, *et al.*, ‘Aktivnyi itdykh *v* rabotosposobnosti pri starenii’, *Gerontologiia i geriatriia 1969–70,* 134.

101
*Ibid.*, l. 187. *Gorodki* was a game like bowls or skittles.

102 TsDAVO, f. 4783, op. 1, d. 232, l. 61. 1969.

103 Gusman and Ianenko listed those who had studied the Sechenov phenomenon since the 1930s. Gusman and Ianenko, ‘Effektivnost’ vliyaniia razlichnykh form aktivnogo otdykha’, 182.

104 TsDAVO, f. 4783, op. 1, d. 52, ll. 73–4; 62.

105 The ‘social contract’ between the state and older people did not apply to all areas, see, for example, Harris, ‘“We Too Want to Live in Normal Apartments”’, 146. Providing benefits in exchange for pensioners continuing to work into retirement became more pronounced under Brezhnev. See G.N. Dzhavadian, ‘Zabolevaemost’ pensionerov i izpol’zovanie ikh trudosposobnosti *v* narodnom khoziastve’, (Dissertation; MZ USSR; All-Union Scientific-Research Institute for Social Hygiene and the Organization of Public Health named for Semashko, Moscow, 1975), 5–7.

106 Sarkizov-Serazini, *op. cit.* (note 92), 7. Interest in ideas about old age were not exclusively Soviet and had roots in pre-revolutionary Russia.

107 N. Sachuk, ‘Science Serves Health: Should a Pensioner Work?’, *Izvestiia*, April 18 (1973), 3, in source: *Current Digest of the Russian Press*, no. 16, Vol. 25, May 16 (1973), 22–3 (accessed via Eastview). On retirement, see also V.D. Shapiro, *Sotsialnaia aktivnost’ pozhilykh liudei v USSR* (Izd. ‘Nauka’, 1983).

108 Lovell, ‘Soviet Russia’s Older Generations’, 219. This was not helped by the ‘moral satisfaction’ argument to keep working and avoid the ‘retirement illness’, see also G.N. Dzhavadian, ‘Zabolevaemost’ pensionerov *i* izpol’zovanie ikh trudosposobnosti v narodnom khoziastve’, 12.

109 WHO. World report on ageing and health. Available at: https://apps.who.int/iris/bitstream/handle/10665/186463/9789240694811_eng.pdf?sequence=1&isAllowed=y, 10, accessed 4 May 2021.

110 Revutskaia *et al.*, ‘Aktual’nye problem’, 10. For further discussion of older people and work, see Aleksandra Brokman, ‘“A Golden Autumn”: Mental Hygiene and Psychoprophylaxis of Old Age in Post-War USSR’, Working paper, 13–15.

111 TsDAVO, f. 4783, op. 1, d. 59, l. 59. See also M.I. Khvilivitskaia and E.V. Kalinina, ‘Zdorov’ye *i* trudosposobnost’ nerabotaiushchikh i rabotaiushchikh pensionerov’, *Gerontologiia i geriatriia 1969–70,* 122–7.

112 TsDAVO, f. 4783, op. 1, d. 59, l. 59.

113 Ages are classified and termed variously in the literature. Elsewhere, based on a 1962 conference of gerontologists in Leningrad, elderly was in the 60–74 age bracket, 75 and older ‘old’ and those aged over 90 years were ‘longlivers’. Chebotarev *et al.*, *Osnovy Gerontologii*, 6. Information on the Leningrad Institute of Expertise for Working Capacity and Organisation of Work for Disabled People has an archive at the Central State Archive of Scientific-Technical Documentation Saint Petersburg (TsGANTD-Spb) – with thanks to the anonymous reviewer for noting this.

114
*Ibid.* In this same discussion, middle age was taken as between 40 and 59/60 years. TsDAVO, f. 4783, op. 1, d. 59, l. 77. Budilin.

115
*Ibid.,* l. 60.

116 N.N. Sachuk, ‘Demograficheskaia *i* sotsial’naia kharakteristika naseleniia starshikh vozrastov’, in *Osnovy Gerontologii* (Moscow: Meditsina, 1969), 511; N.K. Witte and E.I. Stezhenskaia, ‘Gigiena i fiziologiia truda naseleniia starshikh vozrastnykh grupp’, in *Osnovy Gerontologii*, 522.

117 Witte, Stezhenskaia, ‘Gigiena i fiziologiia truda’, 522.

118 Khvilivitskaia, Kalinina, ‘Zdorov’ye i trudosposobnost’, *Gerontologiia i geriatriia 1969–70,* 127. East German media portrayed a similarly positive picture of ageing in the GDR compared to West Germany. James Chappel, ‘On the Border of Old Age: An Entangled History of Eldercare in East Germany’, *Central European History*, 53 (2020), 353–71 (353–5).

119 A.L. Reshetiuk, *et al.*, ‘Gerogigienicheskie problem sovremennogo proizvodstva’, *Vestnik Akademii Medeitsinskikh Nauk SSSR* 10 (1986), 57.

120 TsDAVO, f. 4783, op. 1, d. 59, l. 61.

121
*Ibid.*

122 Harold L. Sheppard, ‘Introduction’, *Toward An Industrial Gerontology: An Introduction to a New Field of Applied Research and Service* (Cambridge, MA: Schenkman Publishing Company, 1970), 2. Witte and Stezhenskaia also discussed international scholars’ research on working conditions for older people. This included Wolfbein’s 1963 findings on age and work in the USA, and referenced the following gerontologists – Fleming, Litt (1957), Clark (1960), Tibbitts (1960), Sheldon (1960), and Eitner (1964) in relation to ‘the rational use of the older age [people] in production’. Witte, Stezhenskaia, ‘Gigiena *i* fiziologiia truda’, 525, 531.

123 Acharkan, ‘Sotsial’no-pravovye mery’, 30. Citing the Yearbook of Labour Statistics, Geneva, 1968.

124 See, for example, Eitner‚ Die entwicklung des alterspezifischen Beschäftigtengrades von Rentnern in der DDR;’ Positive und negative Einflußgroßen auf die Fortführung der Berufstätigkeit im Rentenalter’,’Gerohygienische Aspekte des Arbeitsinsatzes alternder und älterer Menschen,’ *Praktische Gerohygiene*, 98; 99. In the same volume, see also chapters by, inter alia, W. Schüttmann, H. Thiele, and M. Quaas.

125 Daniel Dixon, ‘Some Economic Aspects of Gerontology’, in Sheppard, *Toward An Industrial Gerontology*, 32.

126 For example, see R.M. Belbin, ‘Older People and Heavy Work’, *British Journal of Industrial Medicine* 12 (1955):309–19. Belbin credits Solomon Barbin as the first person to examine the role of older people and industry (in the 1930s), 309. Belbin’s research is referenced in Solomon Barkin, ‘Retraining and Job Redesign: Positive Approaches to the Continued Employment of Older Persons’, in Sheppard, *Toward an Industrial Gerontology*, 23. Also for discussion of older people at work in the UK, see Pat Thane, ‘Ageing and Gerontology in Britain after 1945’, in Susan Grant and Isaac McKean Scarborough (eds), *Geriatrics and Ageing in the Soviet Union: Medical, Social and Political* Contexts (Bloomsbury Academic, forthcoming, 2023). For more on international comparisons, see Isaac McKean Scarborough, ‘A New Science for an Old(er) Generation’.

127 Thane, ‘Ageing and Gerontology’, unpaginated.

128 Sheppard, ‘Introduction’, *Toward an Industrial Gerontology*, 5–6. To this end a two-day research seminar on the matter was organised and an edited volume produced.

129 A.L. Reshetiuk, ‘The Working Ability of Ageing Workers’, in *Gerontology and Geriatrics Education*, Vol. 13, no. 1–2 (1992), 92. Here Reshetiuk made referenced both the United Nations (1982) and the National Council on the Ageing (1972 and 1973).

130
*Ibid.*, 98, 99.

131
*Ibid.* To overcome this, he recommended prioritizing gero-ergonomics as the basis for the ‘gerontological humanization of technology’, Reshetiuk, ‘Working Ability of Ageing Workers’, in *Gerontology and Geriatrics Education*, Vol. 13, no. 1–2 (1992), 100.

132 See, for example, Claire Horton, ‘The big ageing population debate: how can we prepare for an ageing society?’, *The Guardian*, 26 June 2014. Available at: https://www.theguardian.com/society/2014/jun/26/big-ageing-population-debate, accessed October 12, 2020; Neil McFarquhar, ‘Putin Softens Russia Pension Changes, in Rare Bow to Public Pressure’, The New York Times, 29 August 2018. Available at: https://www.nytimes.com/2018/08/29/world/europe/russia-putin-pension.html, accessed 12 October 2020.

133 Sivaramakrishnan, *As the World Ages*, 97–8, citations 98.

134 TsDAVO, f. 4783, op. 1, d. 27, on English language training see ll. 3–4; for an example of international conferences for 1960 see ll. 21–2 and for Chebotarev citation, d. 235, l. 19.

